# Visual body part representation of the lateral occipitotemporal cortex in individuals with autism spectrum disorder: A univariate and multivariate fMRI study

**DOI:** 10.1162/IMAG.a.24

**Published:** 2025-06-05

**Authors:** Yuto Kurihara, Hirotaka Kosaka, Bianca A. Schuster, Ryo Kitada, Takanori Kochiyama, Hidehiko Okazawa, Rieko Osu, Yuko Okamoto

**Affiliations:** Faculty of Human Sciences, Waseda University, Saitama, Japan; Research Center for Child Mental Development, University of Fukui, Fukui, Japan; Department of Child and Adolescent Psychological Medicine, University of Fukui Hospital, Fukui, Japan; Department of Neuropsychiatry, Faculty of Medical Sciences, University of Fukui, Fukui, Japan; Division of Developmental Higher Brain Functions, United Graduate School of Child Development, Osaka University, Kanazawa University, Hamamatsu University School of Medicine, Chiba University, and University of Fukui, Osaka, Japan; Department of Cognition, Emotion and Methods in Psychology, University of Vienna, Vienna, Austria; Graduate School of Intercultural Studies, Kobe University, Hyogo, Japan; Brain activity imaging center, ATR-Promotions, Kyoto, Japan; Biomedical Imaging Research Center, University of Fukui, Fukui, Japan

**Keywords:** ASD, fMRI, RSA, MVPA, LOTC, body-part recognition

## Abstract

The lateral occipitotemporal cortex (LOTC) is a part of the brain network thatprocesses human body recognition. It has been implicated in variousneurodevelopmental conditions, including autism spectrum disorder (ASD). Intypically developing (TD) individuals, functional magnetic resonance imaging(fMRI) studies have shown three distinct response patterns to three categoriesof body parts in the LOTC, namely, action effector body parts, non-effector bodyparts, and facial parts. It is currently unclear whether the similar topologicalorganization of the LOTC is observed in individuals with ASD, and if socialinteraction difficulties in this group may partially result from differences inbody part recognition in this area. In this fMRI study, adults with ASD and TDadults viewed photographs of hands, feet, arms, legs, chests, waists,upper/lower faces, whole bodies, and chairs. Mass univariate analysis showed nodifferences in the LOTC response to whole-body images (relative to images ofchairs) in the bilateral LOTC between adults with ASD and TD adults. Inaddition, there were no group differences in the responses to body parts.Furthermore, multivariate (representational similarity) analyses revealed asignificant similar body part representation organized into three clusters(limbs, torsos, and faces) in the bilateral LOTC between TD adults and thosewith ASD. These results indicate that TD adults and those with ASD havecomparable neural representations within the LOTC for whole bodies and bodyparts.

## 1 Introduction

Human body parts such as faces and limbs convey crucial social information such asidentity, emotions, and intentions. For instance, typically developing (TD) adultsmay easily discern the emotions of a person based on their face or other body parts,which are also rich sources of identity information (Bank et al., 2015;Hock et al., 2017). Autism spectrum disorder (ASD) is aneurodevelopmental condition characterized by social and communicative difficulties,restricted interests, and repetitive patterns of behavior (APA, 2013). Previous behavioral studies have shown thatindividuals with ASD exhibit difficulties in detecting intentions from bodilyactions (Cossu et al., 2012) and inrecognizing facial identity (Weigelt etal., 2012) and emotion (Yeung,2022), which may be related to observed difficulties in socialcommunication. Although these difficulties in recognizing emotions/identity couldarise from differences in the neural representational body parts, the neuralrepresentation of the human body in the brains of individuals with ASD is currentlynot fully understood.

The lateral occipitotemporal cortex (LOTC) is a key node in a wider brain networkthat supports the recognition of body parts and has perceptual and social functions.The perceptual function of the LOTC is the recognition of certain body parts such asa hand or a foot. For instance, seminal functional magnetic resonance imaging (fMRI)studies have found that a part of the LOTC called the extrastriate body area (EBA)is more strongly activated when viewing non-face body parts than when viewing otherobjects such as tools (Downing et al.,2001,^2006^). Contrarily,the LOTC is also involved to distinguish between self and others and to recognizethe intentions of others during social interaction. These LOTC functions areimplicated in action observation and execution (Astafiev et al., 2004;Gazzola & Keysers, 2009;Oosterhof et al., 2012), gestural interaction (Okamoto et al., 2014;Sasaki et al., 2012), and inunderstanding the meaning of actions (Kubiak & Króliczak, 2016;Okamoto et al., 2014;Wurm et al., 2016,^2017^), which provides important information forinteracting with another person.

A number of previous fMRI studies exploring the functions of the LOTC have showndifferences in social functions between TD adults and those with ASD. For instance,adults with ASD showed lower LOTC activation when they were imitated by anotherperson compared to TD adults (Okamoto etal., 2014). Furthermore, a meta-analysis revealed lower activation of theLOTC in individuals with ASD during action understanding and imitation tasks,compared to TD individuals (Yang &Hofmann, 2016). LOTC activation was lower in individuals with ASD than inTD individuals when hand perspective (first- and third-person perspective) was usedto differentiate between self and other bodies (Okamoto et al., 2018). These findings suggest thatindividuals with ASD and those without ASD have differences in various socialfunctions in the LOTC. In contrast, there is limited fMRI study of ASD individualsfocusing on perceptual function in the LOTC. For instance, our previous fMRI studiesusing classical mass-univariate analyses have shown that mean responses to thepresentation of bodies relative to other object in the LOTC are comparable betweenTD adults and adults with ASD (Okamoto etal., 2014,^2017^,^2018^). The study leads the hypothesisthat individuals with and without ASD have similar perceptual functions of theLOTC.

However, more recent studies found different characteristics in perceptual functionin the LOTC; the LOTC has different representation among each body part (Bracci et al., 2015;Orlov et al., 2010).Orlov et al. (2010)found atopographically ordered body part map with separate clusters, demonstrating anobvious preference for different visually presented body parts, such as the upperlimbs or trunk.Bracci et al. (2015)further discovered body parts representation in the LOTC using representationalsimilarity analysis (RSA). In their study, the organization of body partrepresentation within the LOTC was characterized by functional-semantic qualities,forming three distinct groups: action effectors (hands, feet, arms, and legs),non-effector body parts (chest and waist), and facial parts (upper and lower faces).Okamoto et al. (2020)revealedthat, although responses to whole-body images differ between TD children/adolescentsand adults, body part representation in the LOTC of TD children follows afunctional-semantic organization like TD adults. Furthermore, LOTC body partrepresentation was associated with sensory characteristics in TDchildren/adolescents, which is frequently observed in individuals with ASD (Okamoto et al., 2020). If thefunctional organization of ASD individuals is different from that reported for TDindividuals, this may partially explain observed differences in body recognition andsocial interaction between adults with ASD and TD adults. Alternatively, it ispossible that individuals with ASD show similar body representation in the LOTC asTD individuals, and that higher-order functions such as self-other differentiationor social interaction in the LOTC might be associated with social difficulties inASD. However, to the best of our knowledge, no study has tested the functionalsemantic organization of body parts within the LOTC of individuals with ASD.

In the present study, we conducted fMRI to investigate whether body partrepresentation in the LOTC of adults with ASD is organized into the same three bodypart clusters found in TD adults (Bracci etal., 2015;Okamoto et al.,2020). As in our previous study (Okamoto et al., 2020), participants were shownphotographs displaying a hand, foot, arm, leg, chest, waist, upper face (UF), lowerface (LF), whole body, or chair and performed a 1-back task. RSA andclassification-based multivariate pattern analysis (MVPA) were used to investigatebody part representation in the LOTC after revealing LOTC activation using picturesof the whole body (relative to a chair). Here, we explored whether the functionalsemantic organization of body parts in the LOTC of adults with ASD is similar tothat of TD adults or not. Furthermore, our previous study showed that sensoryabnormalities are associated with differences in spatial LOTC organization in TDchildren/adolescents (Okamoto et al.,2020). Hence, we further explored whether spatial body part geometry inthe LOTC was associated with several ASD-related individual traits during adulthood(both ASD and TD). As functional differences between ASD and TD are present duringchildhood but disappear in adulthood (Okamoto et al., 2017), the representation of body parts might becomparable between adults with and without ASD. Alternatively, it may be possiblethat body part representation in the LOTC is different between adults with andwithout ASD, although the overall activation of the human body is similar.

## 2 Methods

### 2.1 Participants

Twenty-three TD adults (mean age ± standard deviation (SD) [range]: 31.0± 10.2 [20–49]) and 23 adults with ASD (mean age ± SD[range]: 30.4 ± 5.31 [20–38]) participated in this study. Allparticipants were diagnosed with ASD by a psychiatrist (H.K.) according to theDSM-5 criteria. General autistic traits, sensory processing characteristics, andintellectual abilities were measured in both TD participants and those with ASD.Specifically, general autistic traits were measured using the SocialResponsiveness Scale (SRS) (Constantino& Gruber, 2005;Kamio et al., 2013) and the Japanese version of the Autism SpectrumQuotient (AQ) (Baron-Cohen et al.,2001;Wakabayashi et al.,2004). Sensory processing characteristics were measured using theSensory Profile (SP) (Dunn,1999;Ito et al.,2013). Intellectual ability was assessed using the Full-ScaleIntelligence Quotient (FSIQ) of the Japanese version of the Wechsler AdultIntelligence Scale-Third Edition (WAIS) (Fujita et al., 2006). The results of the t-test onthe FSIQ scores showed that there was no significant difference between the TDand ASD groups. Handedness was measured in all participants using the EdinburghHandedness Inventory (Oldfield,1971). The study protocol was approved by the Ethics Committees ofthe University of Fukui (Japan) and the Advanced Telecommunications ResearchInstitute International (Japan). This study was conducted in accordance with theprinciples of the World Medical Association Declaration of Helsinki. Writteninformed consent was obtained from each participant after receiving a detailedexplanation of the study.

### 2.2 Magnetic resonance imaging parameters

Using a 3.0-T MR imager (SIGNA PET/MR; GE Healthcare), we obtained functional andstructural volumes. Functional volumes were obtained using T2*-weightedgradient-echo echo-planar imaging (EPI) sequences. Each volume had 53 obliqueslices that were each 3.0 mm thick with a 50% gap between them. With a flipangle (FA) of 90° and an echo time (TE) of 25 ms, the repetition time(TR) between two sequential acquisitions of the same slice was 3,000 ms. Thefield of view was 192 mm × 192 mm. The pixel sizes for the digitalin-plane resolution were 3.0 mm, 3.0 mm, or 64 × 64 pixels.Three-dimensional fast-spoiled gradient-recalled acquisition (TR, 8.464 ms; TE,3.248 ms; FA, 11°; 256 × 256 matrix; voxel size, 1 × 1× 1 mm) was used to create a high-resolution anatomical image.

### 2.3 Experimental setup

Presentation software (Neurobehavioral Systems) implemented on a Windows-baseddesktop computer was used to present visual stimuli and collect responses.Visual stimuli were presented on a liquid-crystal display monitor and viewed bythe participants via a mirror attached to the head coil of an MRI scanner. Headmotion was minimized by placing a comfortable but tight-fitting foam paddingaround the head of each participant.

### 2.4 Task procedure

**Fig. 1.**
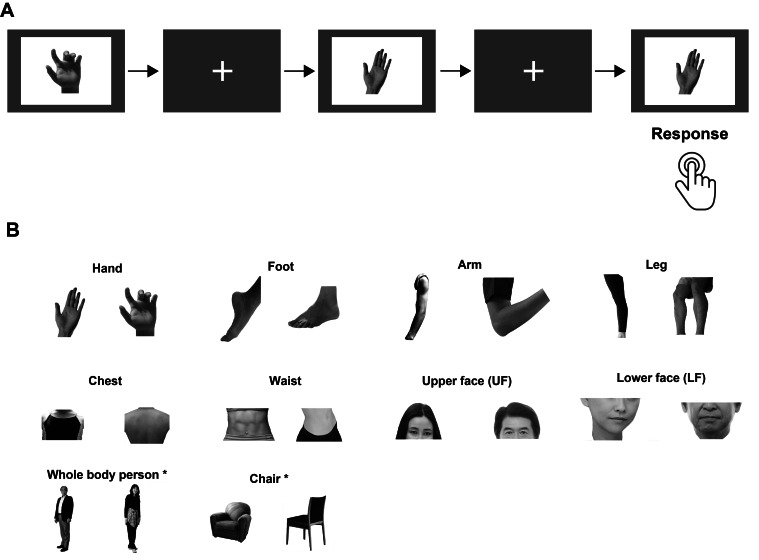
Task procedure. (A) When identical photos were presented in sequence,participants were instructed to press a button. Pictures were shown for500 ms, with a 500 ms interstimulus interval. (B) Examples of stimulifor each condition. Images used to identify the region of interest aredenoted by asterisks (*).

The same task as in our previous study (Okamoto et al., 2020) (Fig.1) was utilized in this study. Specifically, the participantscompleted four runs during which they looked at grayscale images comprising 10different conditions (whole bodies, hands, feet, arms, legs, chests, waists,UFs, LFs, and chairs). Images of each condition, except that for the chaircondition, included the entire bodies or body parts of 11 men, 11 women, 11boys, and 11 girls. The chair conditions included 11 different types of chairs.Thus, 440 images were presented for each condition. All images were changed togreyscale with a white background color and a matrix size of 800 × 600pixels using Adobe-Photoshop software (Adobe System Inc.). Each run consisted of20 task blocks (10 conditions × 2 repetitions = 20 blocks). Ineach block, 12 pictures were presented for 500 ms, with a 500 ms interstimulusinterval. Thus, each block lasted 12 s. The conditions were arranged in apseudo-random order. A fixation-only baseline period was inserted before the1^st^block (27 s), after the 5^th^, 10^th^, and15^th^blocks (12 s), and after the 20^th^block (15 s).Identical pictures were presented in sequence one per block, and participantswere asked to complete a 1-back task in which they were required to press abutton upon viewing two identical pictures in sequence.

### 2.5 Data analysis

#### 2.5.1 Demographic data analysis

Age, FSIQ, SRS, AQ, low registration score, sensory seeking score, sensorysensitivity score, and sensation avoidance score between the ASD and TDgroups were compared using Welch’s t-test. All*p*-values were corrected using Bonferroni correction.

#### 2.5.2 Behavioral data analysis of the 1-back task

The correct response ratio, false alarm ratio, and reaction times for thetarget stimuli in the 1-back task, as well as number of responses for allstimuli were calculated for each participant. Correct response and falsealarm ratios, and number of responses across all conditions were initiallycompared between TD and ASD groups using Welch’s t-test in R (https://www.r-project.org/). Additionally, we explored whethercorrect response ratios and reaction times differed by condition using atwo-way analysis of variance (ANOVA) with the factors body parts (wholebodies, chairs, hands, feet, arms, legs, chests, waists, UFs, and LFs) andgroups (TD and ASD) using R.

#### 2.5.3 fMRI data analysis

##### 2.5.3.1 Preprocessing

The first five volumes of each run were discarded due to unsteadymagnetization. The remaining volumes were analyzed using StatisticalParametric Mapping software (SPM12; Wellcome Department of ImagingNeuroscience) (Friston et al.,1994) implemented in MATLAB 2021b (MathWorks). All functionalimages were initially realigned. The high-resolution anatomical imageswere then co-registered to the mean image of the realigned functionalimages and normalized to a tissue probability map that had already beenfitted to the Montreal Neurological Institute (MNI) space via asegmentation–normalization procedure. The parameters from thesegmentation-normalization process were then applied to all functionalimages, which were resampled to a final resolution of 2 × 2× 2 mm^3^. A region of interest (ROI) analysis wasperformed using normalized unsmoothed images. To localize the whole-bodysensitive region in the LOTC, normalized fMRI images were filtered usinga Gaussian kernel of 8 mm (full width at half maximum) in the x-, y-,and z-axes.

##### 2.5.3.2 Statistical analyses

The region that responded to viewing the whole body (i.e., thewhole-body-sensitive region) in the LOTC was initially localized foreach participant. We then conducted univariate and multivariate analyseswithin this whole-body-sensitive region to examine the activationpatterns upon viewing different body parts. In both analyses, activationpatterns in LOTC between TD and ASD participants were compared.

###### 2.5.3.2.1 ROI definition for the whole-body sensitive region

Classical mass-univariate analysis was conducted at two levels todefine whole-body-sensitive ROIs in the LOTC. In the first-levelsingle-subject analysis, a general linear model was fitted to thefMRI data of each participant (Friston et al., 1994;Worsley & Friston,1995). The blood-oxygen level-dependent signal wasmodeled using boxcar functions convolved with a canonicalhemodynamic response function. Each run included one regressor foreach of the 10 conditions (whole bodies, chairs, hands, feet, arms,legs, chests, waists, UFs, and LFs) and six regressors ofmotion-related parameters (three displacements and three rotationsobtained by the rigid-body realignment procedure). The time seriesof each voxel was high pass filtered at 1/128 Hz. Serialautocorrelation was estimated from a collection of pooled activevoxels using the constrained maximum likelihood approach, assuming afirst-order autoregressive model, and was used to whiten the data(Friston et al.,2002). Global signal scaling, such as scanner gainchanges, is used to eliminate global confusion. The contrastestimates of whole bodies versus chairs for each participant werecompared using linear contrasts.

Thereafter, a second-level group analysis was performed on contrastimages (whole bodies vs. chairs) from the first-level analyses, andwhole-body-sensitive regions (whole-body vs. chair) in eachhemisphere were identified within each group. The resulting set ofvoxel values for each contrast constituted SPM{t}. The statisticalthreshold of SPM{t} was set at*p*< 0.05 tocorrect for multiple comparisons at the cluster level over theentire brain (family-wise error), with a height threshold of*p*< 0.001. In addition, to explorepotential differences across the whole brain between TD and ASD, weperformed a classical analysis using an uncorrected*p*-value threshold of 0.05, as well as aBayesian 2nd-level analysis (Han & Park, 2018).

Subsequently, the activation of the whole-body sensitive regionbetween TD participants and those with ASD was compared. We examinedfour variables using individual ROI analyses in the following order:ratio of participants exhibiting a category-sensitive reaction,extent of activation (i.e., total number of voxels over a threshold)for participants exhibiting this response, peak coordinate positionfor persons displaying activation, and activation pattern among fourobject categories at individual peak coordinates for thoseexhibiting this response. To avoid circular analysis, an8-mm-diameter sphere centered on the coordinates of the originalstudy was utilized (Bracciet al., 2015), which was converted from Talairach to MNIcoordinates (Lancaster etal., 2007). The statistical threshold was set at*p*< 0.01, uncorrected for multiplecomparisons (Okamoto etal., 2020). To compare the ratios of participants showingactivation and the size of activation between TD individuals andthose with ASD, a$\chi^{2}$test and a two-sample t-test were conducted in R, respectively.

###### 2.5.3.2.2 ROI analysis for eight body parts

ROI analyses were based on the beta images (parameter estimates foreach voxel) resulting from the single-subject mass-univariatewhole-brain analysis performed on unsmoothed data. To rule out thepossibility that group differences in ROI volumes mask anybetween-group differences in activation measures, the meanactivation of the ASD and TD groups (contrast of whole bodies vs.chairs) was used.

**Univariate regional average activation analysis:**Meanbeta estimates of eight body parts relative to baseline wereextracted from the ROIs in each hemisphere for each participant toassess the overall activation intensity of the ROIs. R software wasused to perform a two-way ANOVA on both body parts and groups.

**Representational similarity analysis**: Therepresentational geometry of the neural population code wasevaluated using correlation-based RSA (Bracci et al., 2015;Haxby et al., 2001;Kriegeskorte &Kievit, 2013). Within the whole-body sensitive region ineach hemisphere, parameter estimates of eight body parts (hands,feet, arms, legs, chest, waist, UFs, and LFs) were compared with thefixation-only baseline of each voxel for each run. Aneight-condition × eight-condition dissimilarity matrix (1 -r) for each ROI was built for each participant based on thecorrelation coefficient r between the parameter estimates of the oddand even runs. For each group, a mean dissimilarity matrix wasgenerated, and multidimensional scaling (MDS) was used to visualizethe similarity structures using the MATLAB function mdscale.

To examine whether the spatial activation patterns in the LOTC wereorganized according to the three categories (action effector bodyparts, non-effector body parts, and face parts) in each group, weutilized a hypothesis-driven approach based on previous studies(Bracci et al.,2015;Okamotoet al., 2020). For each group, an analysis of similarity(ANOSIM) (Clarke,1993;Legendre& Legendre, 1998a,1998b) was performed using the FathomToolbox (Jones,2017) in MATLAB. ANOSIM is a hypothesis-drivennon-parametric statistical test frequently employed in ecology todetermine whether the similarity between categories is greater thanor equal to the similarity within categories. According to ANOSIM,which provides a dissimilarity index (R-value) ranging from -1 to+1, a result near 1.0 indicates a strong grouping or highseparation of body part categories. The statistical tests on the Rvalues included a complete permutation test comparing thehypothesized model ([1] hand, arm, leg, and foot; [2] chest andwaist; and [3] UF and LF) with 419 alternative models ([1] arm, leg,chest, and waist; [2] UF and LF; [3] foot, hand, and other possiblepatterns), totaling 420 patterns. This statistical method assessesthe relevance of the hypothesized model compared to alternatives,making ANOSIM more suitable than approaches like hierarchicalclustering for this study.

We further examined the comparability of spatial activation patternsin the LOTC between the TD and ASD groups. Initially, we utilizedthe Mantel test, which measures the correlation between twodissimilar matrices (Legendre & Legendre, 1998a,1998b;Mantel, 1967). TheMantel test employs the Pearson product-moment correlationcoefficient (R), which ranges from -1 to 1. An R-value of ±1indicates a perfect positive or negative association, respectively.The null hypothesis (H0) assumes that there is no significantrelationship between the two matrices, meaning the elements of onematrix are independent of those in the other. Under this hypothesis,any observed correlation is attributed to random chance. Conversely,the alternative hypothesis (H1) asserts that there is a significantcorrelation between the matrices, suggesting a systematicrelationship between elements that cannot be explained by randomvariation alone. Therefore, rejecting H0 in favor of H1 implies thatthe two matrices are significantly similar.

Regarding individual variability, correlation coefficients weredetermined between the dissimilarity matrix of each participant andthe mean dissimilarity matrix of their group, as well as between thedissimilarity matrix of each participant and the mean dissimilaritymatrix of the other group. When correlating the dissimilarity matrixof a particular participant with those in the participant’sgroup, the group dissimilarity matrix was calculated by excludingthe data for that participant. Four correlation coefficients(correlation between a participant with TD and the TD group, thecorrelation between a participant with TD and the ASD group, thecorrelation between a participant with ASD and the TD group, and thecorrelation between a participant with ASD and the ASD group) werecalculated. The correlations were normalized using Fisher’s Ztransformation. The type of correlation (within-groupcorrelation/between-group correlation) and group (TD/ASD) were thencompared using two-way ANOVA with R.

Finally, we investigated whether spatial body part geometry in theLOTC was associated with several ASD-related individual traits.First, the R-value of the ANOSIM for the LOTC ROIs of eachparticipant was generated and utilized as a measure of spatial bodypart organization. For each hemisphere, a Spearman’s rhocorrelation analysis was performed between R values and scoresmeasuring autistic traits (SRS and AQ total scores), sensoryprocessing characteristics (SP low registration, SP sensory seeking,SP sensory sensitivity, and SP sensation avoidance), age, andintellectual ability (FSIQ). R software was used for this analysis,and Bonferroni correction was performed.

**Classification-based multivariate pattern analysis:**Inaddition to the RSA, the varied spatial activation patterns of theLOTC between categories (action effector [hands, limbs, arms, feet],faces [UFs, LFs], and non-effector body parts [chest, waist]) wereconfirmed using a support vector machine (SVM)-based classificationanalysis. For this, the Decoding Toolbox (TDT) (Hebart et al., 2014)implemented in SPM12 was utilized. The beta values for the featurevectors and a linear SVM classification with a leave-one-run-outcross-validation approach were used. More specifically, featurevectors were created in three of the four runs and tested in theremaining one. We repeated the process four times, changing the testrun. TDT offers a template script for unbalanced data (i.e.,decoding template unbalanced data), which was used during thecross-validation iteration because this study used an unbalanceddesign (i.e., the number of conditions varied within categories). Intotal, 100 bootstrap samples were used in the analysis. For the LOTCROIs, the classification accuracy above chance level was determinedto assess the classification performance. A one-sample t-test in Rwas used to determine if the decoding accuracies were higher thanexpected by chance (33.3%), and a two-sample t-test was used tocompare the decoding accuracies between the two groups (TD and ASD).Furthermore, comprehensive permutation testing was used to confirmwhether the spatial activation patterns in the LOTC could beclassified into one of three classes in each group: action effector,face, or non-effector. The condition labels of each class (orcategory) were permuted to produce an empirical null distribution,which was used to estimate the likelihood of the original(hypothesized) labeling. For the three-class categorization, thisresulted in a statistically significant*p*-value.All 420 feasible possibilities were investigated using a permutationprocess.

## 3 Results

### 3.1 Demographic data

There were significant differences in SRS, AQ, and one sub-scale of sensoryprofile (Sensory seeking) between the TD and ASD groups, according to thetwo-sample Student’s t-test (Table 1). In contrast, there were no significant differences in age,FSIQ, and other three SP sub-scales between the TD and ASD groups (Table 1).

**Table 1.** Demographic data.

	TD group	ASD group	*t*	*p* (uncorrected)	*p* (corrected)	Cohen’s *d*
N	23	23	-	-	-	-
Age (years)	31.0 ± 10.2	30.4 ± 5.3	0.26	0.815	1.000	0.069
Handedness (right/left/both)	20/2/1	23/0/0	-	-	-	-
FSIQ	111.0 ± 14.4	109.0 ± 12.0	0.43	0.667	1.000	0.131
SRS score	54.9 ± 21.2	106.0 ± 28.9	-6.82	<0.001***	<0.001***	-2.012
AQ total score	16.0 ± 7.0	33.0 ± 5.0	-9.35	<0.001***	<0.001***	-2.756
SP						
Low registration score	27.4 ± 7.5	33.7 ± 8.7	-2.63	0.012*	0.093	-0.776
Sensory seeking score	38.1 ± 6.4	30.7 ± 6.6	3.81	<0.001***	0.003**	1.124
Sensory sensitivity score	33.9 ± 6.9	40.2 ± 9.7	-2.56	0.014*	0.113	-0.754
Sensation avoiding score	34.7 ± 6.3	40.9 ± 11.4	-2.28	0.028*	0.222	-0.671

*Note:*Handedness was assessed using the EdinburghHandedness Inventory (Oldfield, 1971). FSIQ, full-scale intelligence quotientof the Wechsler Adult Intelligence Scale-Third Edition (WAIS-III(Wechsler,1997); SRS, Social Responsiveness Scale (Constantino & Gruber,2005;Kamio etal., 2013); AQ, autism spectrum quotient (Baron-Cohen et al.,2001); SP, sensory profile (Dunn, 1999;Ito et al., 2013); TD, typicallydeveloping; and ASD, Autism spectrum disorder. All*p*-values were adjusted using the Bonferronicorrection (*p< 0.05,**p< 0.01,***p< 0.001).

### 3.2 Behavioral results

**Fig. 2.**
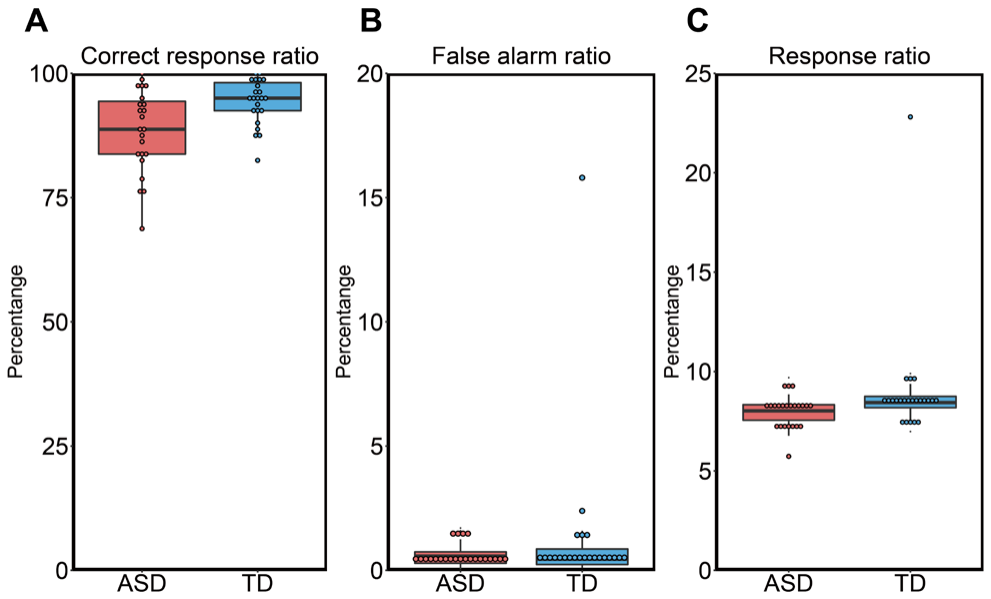
Behavioral performance of the 1-back task. (A) The correct response ratiofor target stimuli, (B) the false-alarm ratio, and (C) the responseratio for all stimuli are shown. TD, typically developing; ASD, autismspectrum disorder.

For the 1-back task, the overall percentage (%) of correct responses to targetstimuli (correct response ratio) was significantly greater in the TD group thanin the ASD group, according to the two-sample Welch’s t-test(*t*_34.15_= -2.92,*p*= 0.006, Cohen’s*d*= -0.861;Fig. 2A). In contrast, there wereno group differences in the false alarm ratio(*t*_23.09_= -1.02,*p*= 0.318, Cohen’s*d*= -0.301;Fig. 2B) or response ratio for allstimuli (*t*_25.09_= -1.69,*p*= 0.104, Cohen’s*d*= -0.497;Fig. 2C).

Furthermore, we explored group differences in reaction time and correct responseratio using a two-way ANOVA on body parts and groups. The analysis of reactiontime revealed a significant main effect of body part(*F*_1,396_= 3.74,*p*< 0.001,$\eta_{p}^{2}$= 0.078) (Supplementary Fig. S1A). In contrast, there was no significant maineffect of group (*F*_1,396_= 1.21,*p*= 0.280,$\eta_{p}^{2}$= 0.027) and no interaction of group and body parts for reaction time(*F*_1,396_= 1.80,*p*= 0.066,$\eta_{p}^{2}$= 0.039). The posthoc analysis of body parts showed significantdifferences in reaction time between whole body and chests, legs, waists, feet,and arms, as well as between chairs and feet (whole body < chest,*p*< 0.001; whole body < leg,*p*< 0.001; whole body < waist,*p*= 0.002; chair < foot,*p*= 0.005; whole body < foot,*p*= 0.026;whole body < arm,*p*= 0.032). The two-way ANOVAon correct response ratio showed a significant main effect of group(*F*_1,396_= 10.38,*p*= 0.002,$\eta_{p}^{2}$= 0.191) (Supplementary Fig. S1B). However, there was no significant maineffect of body part (*F*_1,396_= 1.20,*p*= 0.293,$\eta_{p}^{2}$= 0.027) and no interaction of group and body part in the correctresponse ratio (*F*_1,396_= 0.78,*p*= 0.639,$\eta_{p}^{2}$= 0.017). Collectively, the correct response rate of participants withASD was lower than that of TD participants, regardless of the condition.

### 3.3 fMRI results

#### 3.3.1 Head motion

The head motion during scanning was initially confirmed in each group. Themean framewise displacement (FD) (Power et al., 2012) for each participant was calculated andcompared between ASD and TD groups. There was no significant difference inhead motion between ASD and TD groups (ASD group: mean ± SD =0.102 ± 0.077, TD group: mean ± SD = 0.141 ±0.139;*t*_34.34_= -1.17,*p*= 0.250, Cohen’s*d*= -0.345).

#### 3.3.2 The whole-body-sensitive region in the LOTC

**Fig. 3.**
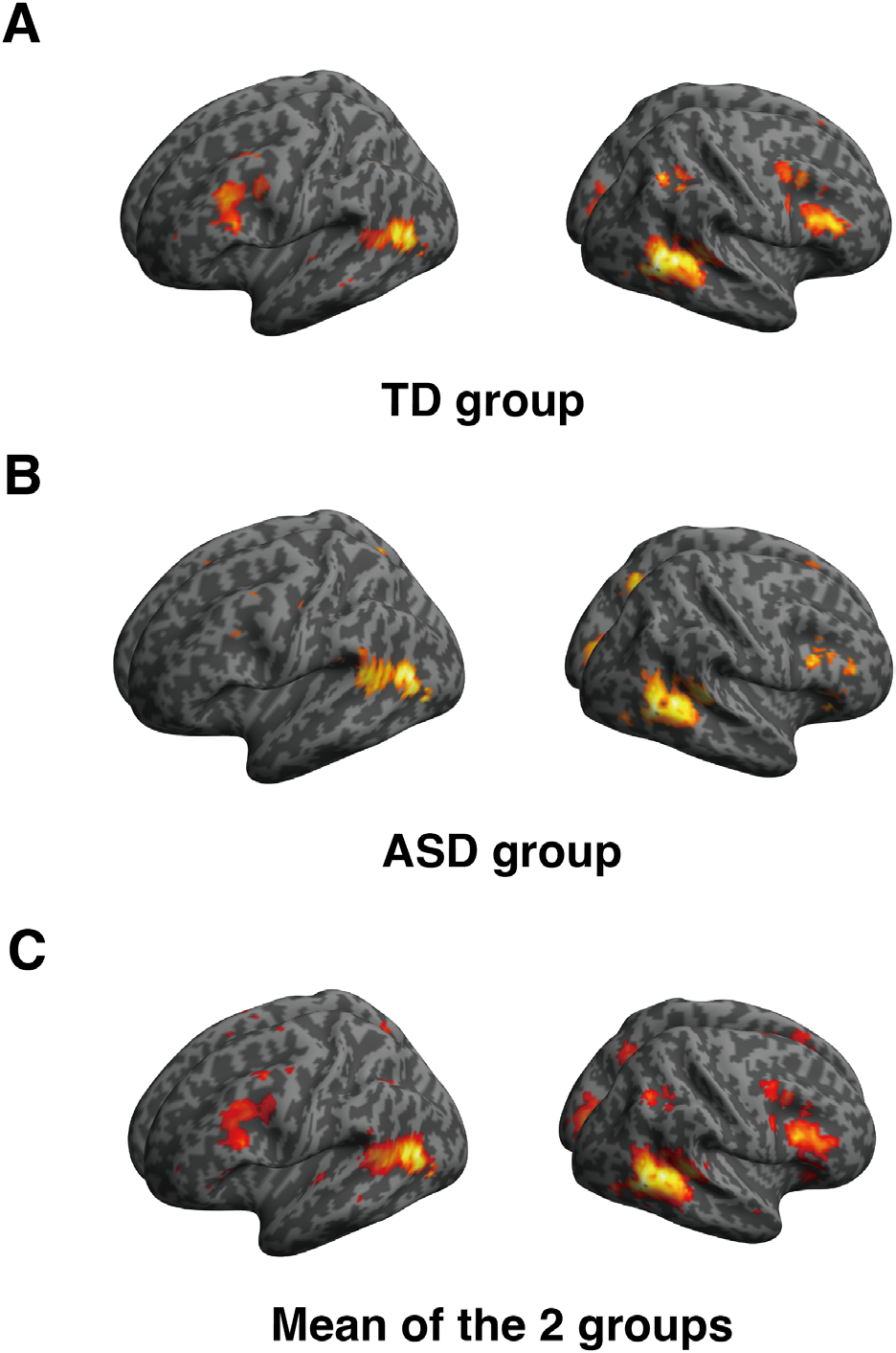
Whole-brain analysis: Whole-body sensitive regions in the TD and ASDgroups. Whole-brain person-sensitive regions (whole-body vs. chair)in (A) the TD group and (B) the ASD group. (C) Mean of the twogroups superimposed on a T1-weighted MRI. The threshold for theheight of the activation was set at*p*<0.001, while the size of the activation was set at*p*< 0.05 with multiple comparisoncorrections using family wise error. TD, typically developing; ASD,autism spectrum disorder.

The whole-body-sensitive region in the LOTC was depicted for each group usingthe contrast of whole bodies versus chairs. We examined differences inactivation between the ASD and TD groups. In the group analysis, activationwas found in the bilateral LOTC in both the ASD and TD groups (Fig. 3A, B). We then comparedthe activation intensity between the ASD and TD groups; there was nosignificant difference.Figure3Cshows the mean activation of the ASD and TD groups, which wereused for subsequent ROI analyses.

On the other hand, we checked the cluster groups that represent thedifference of activities between TD and ASD when the uncorrected*p*-value threshold was set to 0.05 (SupplementaryFig. S2). Similarly, we checked the cluster groups that representthe difference of activities between TD and ASD when 2nd-level Bayesiananalysis is performed (Effect size > 0.2) (SupplementaryFig. S3). Even when the threshold was lowered in these additionalanalyses, no notable differences emerged in the vicinity of the LOTCregion.

For individual analyses, the size of whole-body sensitive activation and theratio of participants showing whole-body sensitive regions in the LOTCbetween the ASD and TD groups were compared (Table 2). In the left hemisphere, 21 out of23 participants in the TD group (91%) and 21 out of 23 participants in theASD group (91%) exhibited activation; there were no group differences(χ_12_= 0,*p*= 1,*Cramer’s V*= 0). There was no significantdifference in the activation size (*t*_32.41_= 1.27,*p*= 0.205, Cohen’s*d*= 0.340) using Welch’s t-test. In theright hemisphere, 23 out of 23 participants in the TD group (100%) and 22out of 23 participants in the ASD group (96%) exhibited activation; therewere no group differences (χ_12_= 0,*p*= 1,*Cramer’s V*= 0.149). In addition, there was no significant difference betweenthe TD and ASD groups in terms of activation size(*t*_37.88_= 0.979,*p*= 0.334, Cohen’s*d*= 0.289;Table 2). Therefore, theactivation size and ratio of participants in the whole-body sensitive regionwere highly similar in the ASD and TD groups.

**Table 2.** Individual analysis: Whole-body-sensitive region in ASD and TDgroups.

		Peak Coordination					
		x	y	z	Size ( $mm^{3}$ )	Ratio and Percentage	
L.LOTC	ASD	-47.9 ± 2.4	-69.9 ± 2.8	3.9 ± 2.8	20.1 ± 22.6	21/23	91%
	TD	-47.5 ± 2.9	-69.6 ± 2.0	4.0 ± 3.0	33.0 ± 38.4	21/23	91%
R.LOTC	ASD	51.6 ± 2.1	-68.2 ± 2.0	6.5 ± 3.4	52.9 ± 48.56	22/23	96%
	TD	50.4 ± 2.3	-67.7 ± 2.9	5.5 ± 2.9	71.7 ± 75.10	23/23	100%

*Note:*The LOTC was established by 8 mm-diameterspheres centered on the peak coordinates of the brain regionrepresenting the whole body as opposed to a chair (Bracci et al.,2015) (height threshold*p*<0.01). The mean ± standard deviation of the mean ispresented for each value. L, left; R, right; TD, typicallydeveloping; ASD, autism spectrum disorder; LOTC, lateraloccipitotemporal cortex.

#### 3.3.3 ROI analysis

##### 3.3.3.1 Univariate regional average activation analysis

**Fig. 4.**
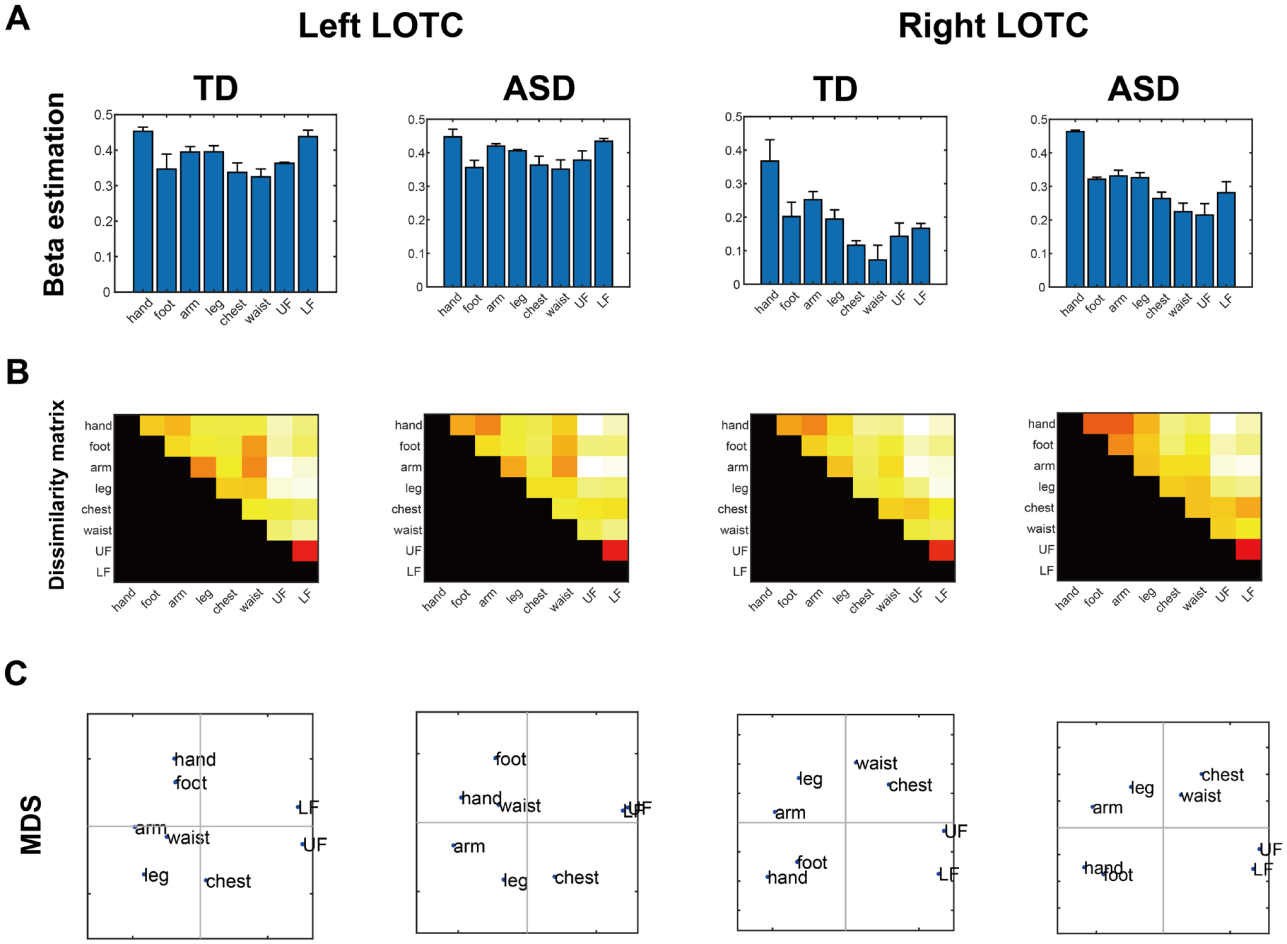
Region-of-interest analysis for TD and ASD groups. (A) Mean betaestimates of each body part relative to the baseline. The errorbars show the SEM. (B) Heatmaps represent dissimilarity matrices(1 − r). (C) MDS data are depicted. LOTC, lateraloccipitotemporal cortex; UF, upper face; LF, lower face; TD,typically developing; ASD, autism spectrum disorder; MDS,multidimensional scaling; SEM, standard error of the mean.

To assess the overall activation intensity for each body part, the meanbeta estimates of eight body parts (hands, feet, arms, legs, chest,waist, UFs, and LFs) compared with the baseline were extracted from eachROI (Fig. 4A). A two-wayANOVA on body part and group revealed a significant main effect of bodyparts (right:*F*_7,308_= 16.68,*p*< 0.001,$\eta_{G}^{2}$= 0.047; left:*F*_7,308_= 6.65,*p*< 0.001,$\eta_{G}^{2}\;$= 0.018). There was no significant main effectof group (right:*F*_1,44_= 1.33,*p*= 0.255,$\eta_{G}^{2}$= 0.026; left:*F*_1,44_= 0.01,*p*= 0.937,$\eta_{p}^{2}\;$= 0.001) or interaction of group and body part(right:*F*_7,308_= 0.54,*p*= 0.802,*$\eta_{p}^{2}$*= 0.002; left:*F*_7,308_= 0.09,*p*= 0.999,$\eta_{p}^{2}\;$= 0.0003). Post-hoc pairwise comparisons withBonferroni correction revealed significant differences between the handand other body parts, arm versus waist, leg versus waist, and footversus waist for the right hemisphere, and hand versus foot, hand versuswaist, hand versus chest, waist versus LF, chest versus LF, leg versuswaist, UF versus LF, hand versus UF, and arm versus waist for the lefthemisphere (all*p*values <0.05). Even when usingpercent signal change, similar results were obtained to those using thebeta value (Supplementary Fig. S4, see SupplementalInformation). Thus, the overall activation intensity of the bilateralLOTC when viewing each body part was comparable between TD individualsand those with ASD.

##### 3.3.3.2 Representational similarity analysis

The representational geometry of body parts in the whole-body-sensitiveregions was investigated (Fig.4B, C). ANOSIM revealed that the spatial activation patternin the whole-body sensitive region was well characterized by threedistinct clusters in both the TD (*R*= 0.900,*p*= 0.005 for the right hemisphere;*R*= 0.675,*p*= 0.024for the left hemisphere) and ASD groups (*R*=0.925,*p*= 0.005 for the right hemisphere;*R*= 0.675,*p*= 0.017for the left hemisphere). Therefore, the action effectors (hand, foot,arm, and leg), faces (upper face and lower face), and non-effector bodyparts (chest and waist) were clustered separately in the bilateral LOTCin the ASD and TD groups.

We then investigated if the dissimilarity matrices were comparablebetween the ASD and TD groups. The Mantel test revealed that thedissimilarity matrices of both groups were highly similar(*R*= 0.925,*p*<0.001 for the right hemisphere:*R*= 0.892,*p*< 0.001 for the left hemisphere).Furthermore, we performed Bayesian linear regression analysis on theTD/ASD dissimilarity metrices body parts representations. The resultsshowed extremely large Bayes factors (BF > 10^4^),providing decisive evidence that the slope (β) is non-zero inboth the left and right LOTC. These results strongly support thealternative hypothesis and indicate positive similarity between TD andASD body part representations in left/right LOTC (seeSupplementalInformation).

**Fig. 5.**
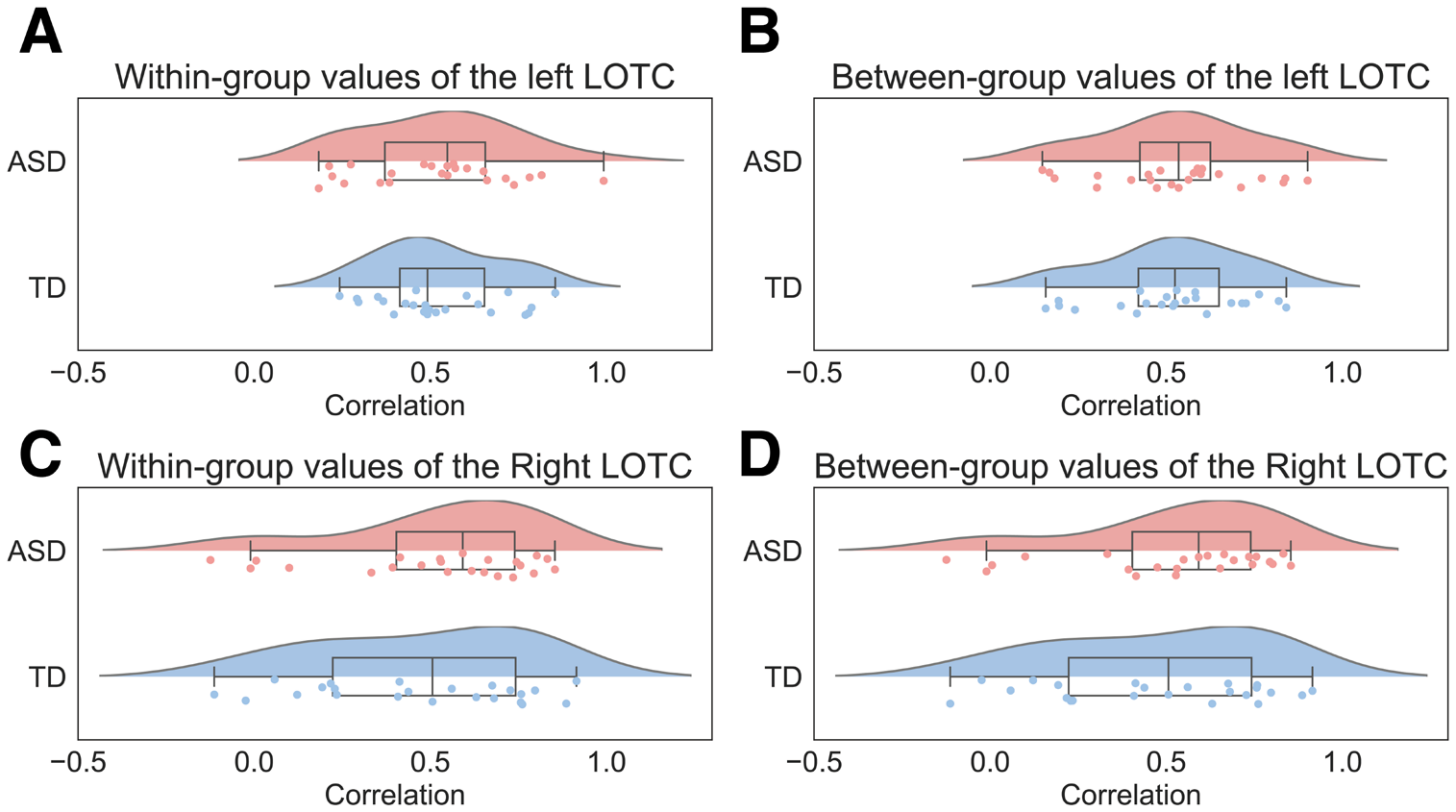
Correlation coefficients between the dissimilarity matrix of eachparticipant and the average dissimilarity matrix within andbetween groups. LOTC, lateral occipitotemporal cortex.Correlation coefficients of (A) within-group values in the leftLOTC, (B) between-group values in the left LOTC, (C)within-group values in the right LOTC, and (D) between-groupvalues in the right LOTC are shown. LOTC, lateraloccipitotemporal cortex.

Among the correlation of each participant’s dissimilarity matrixand group dissimilarity matrix, a two-way ANOVA (correlation type[within-group correlation/between-group correlation] and group [ASD/TD])on correlation coefficients in the left LOTC revealed no significantmain effect of correlation type (*F*_1,44_= 0.58,*p*= 0.449,$\eta_{p}^{2}$= 0.013;Fig. 5A,B) or group (*F*_1,44_= 0.15,*p*= 0.704,$\eta_{p}^{2}$= 0.003;Fig. 5A,B) and no interaction of correlation type and group(*F*_1,44_= 0.20,*p*= 0.657,$\eta_{p}^{2}$= 0.005;Fig. 5A,B). In the right LOTC, there was no significant main effectof correlation type (*F*_1,44_= 3.67,*p*= 0.062,$\eta_{p}^{2}$= 0.077;Fig. 5C,D), main effect of group (*F*_1,44_= 0.24,*p*= 0.629,$\eta_{p}^{2}$= 0.005;Fig. 5C,D), or interaction of correlation type and group(*F*_1,44_= 0.72,*p*= 0.402,$\eta_{p}^{2}$= 0.016;Fig. 5C,D).

We further investigated the representational geometry (within-groupvalue) in the LOTC in relation to individual ASD-related traits in theASD and TD groups. No significant correlation was found for any of theindividual trait measures in the TD group or for the integration of boththe ASD and TD groups (Table3).

**Table 3.** Spearman correlations between ANOSIM R and each score ofquestionnaires.

	(Rho)							
Spearman Correlation	Age	FSIQ	AQ	SRS	SP1	SP2	SP3	SP4
All								
R (Left LOTC)	0.096	-0.094	-0.020	-0.178	-0.170	-0.158	-0.127	0.029
R (Right LOTC)	0.128	0.094	0.106	0.141	0.066	-0.243	0.170	0.162
ASD								
R (Left LOTC)	0.311	0.159	0.215	-0.159	-0.185	0.022	-0.298	-0.097
R (Right LOTC)	0.104	0.094	0.474	0.421	0.092	-0.265	0.140	0.103
TD								
R (Left LOTC)	-0.070	0.304	-0.270	-0.326	-0.187	-0.358	-0.099	0.117
R (Right LOTC)	0.117	0.106	-0.234	-0.170	-0.037	-0.194	0.040	0.108

*Note:*FSIQ, full-scale intelligence quotientof the Wechsler Adult Intelligence Scale-Third Edition(WAIS-III); SRS, social responsiveness scale; AQ, autismspectrum quotient; SP1, Low registration score of thesensory profile: SP2, Sensory-seeking score of the sensoryprofile; SP3, Sensory sensitivity score of the sensoryprofile; SP4, Sensation-avoiding score of sensory profile;TD, typically developing; ASD, autism spectrum disorder;LOTC, lateral occipitotemporal cortex.

In summary, the spatial representation of each body part in thewhole-body sensitive region of the LOTC was organized into threeclusters (action-effector body parts, faces, and non-effector bodyparts) in both groups and was highly similar between the ASD and TDgroups. Furthermore, we did not observe any significant correlationsbetween representational geometry and ASD-related traits.

##### 3.3.3.3 Classification-based multivariate pattern analysis in the LOTC

**Fig. 6.**
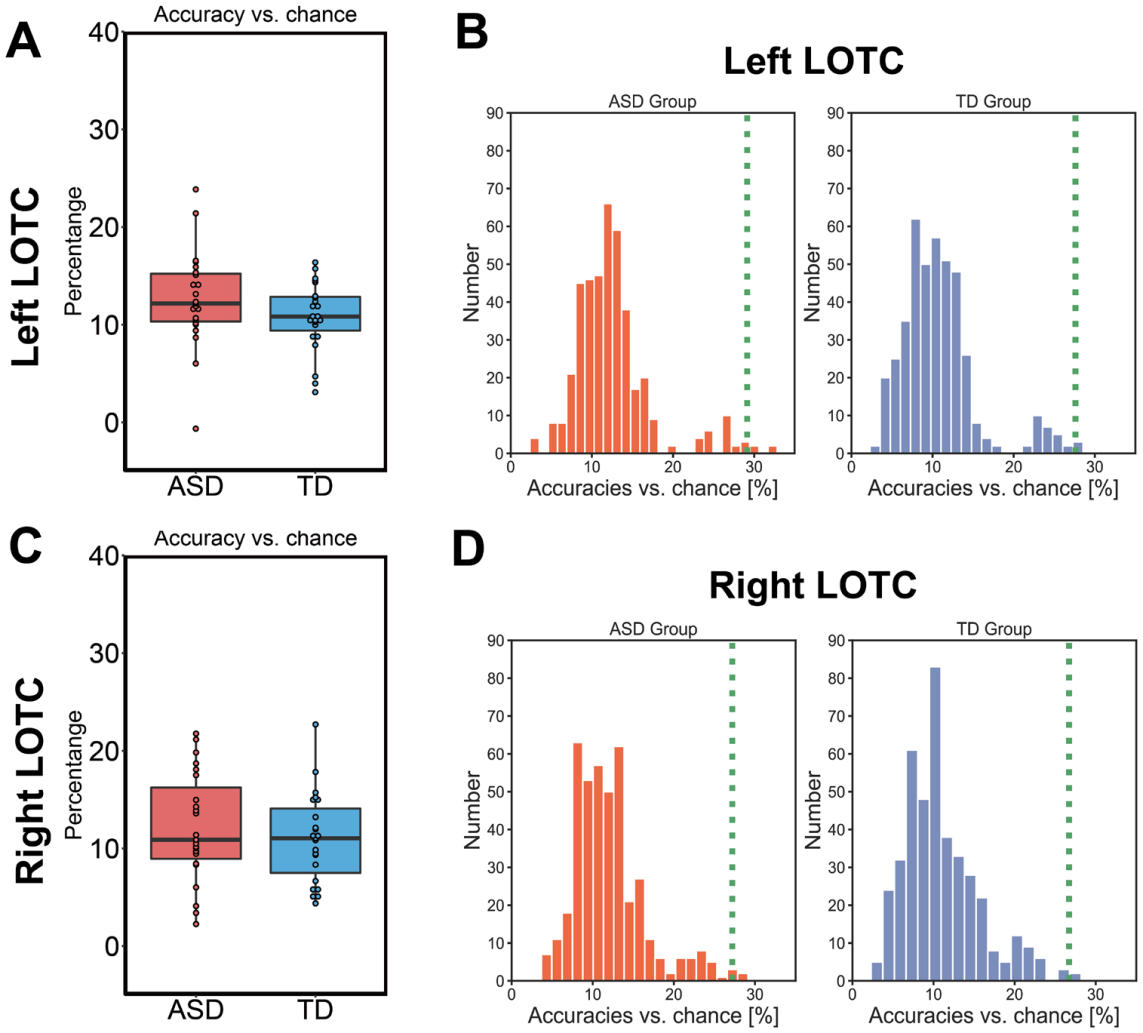
Classification analysis using support vector machine. Zeropercent indicates a chance level (33.3%). (A, C) Classificationaccuracies versus chance (33.3%) in the left and right LOTC areshown. (B, D) Histograms of classification accuracies versuschance determined using the 419 possible combinations ofcircumstances. The histograms show the null distribution ofclassification accuracies (vs. 33.3 chance level) for each ofthe 419 permutative combinations of three clusters. The greendashed lines show original classification accuracies for the oneremaining combination based on the three categories: actioneffectors, faces, and non-effector body parts. LOTC, lateraloccipitotemporal cortex; TD, typically developing; ASD, autismspectrum disorder.

Finally, the spatial activation patterns were confirmed to depend on thecategories (i.e., action effector body parts, faces, and non-effectorbody parts) using a classification-based multivariate analysis.Classification accuracies were above chance level in both the ASD (righthemisphere:*t*_22_= 10.38,*p*< 0.001, Cohen’s*d*= 2.165; left hemisphere:*t*_22_= 12.31,*p*< 0.001, Cohen’s*d*= 2.570;Fig. 6A, C) and TD (right hemisphere:*t*_22_= 11.50,*p*< 0.001, Cohen’s*d*= 2.300; lefthemisphere:*t*_22_= 14.73,*p*< 0.001, Cohen’s*d*= 3.072;Fig. 6A,C) groups, and there were no significant differences inclassification accuracy between the groups (right hemisphere:*t*_42.38_= 0.73,*p*= 0.467, Cohen’s*d*= 0.216; lefthemisphere:*t*_39.83_= 1.45,*p*= 0.154, Cohen’s*d*= 0.429). In both the ASD and TD groups, the spatial activationpatterns in the LOTC were well characterized by three clustersrepresenting action effectors, faces, and non-effector body parts (Fig. 6B, D). Thus, weconfirm that body part representation in the bilateral LOTC was highlysimilar between the ASD and TD groups, further supporting the RSAresults.

## 4 Discussion

In this study, we investigated whether body part representations in the left/rightLOTC of adults with ASD are organized into the same three body part clusters foundin TD adults. In the behavioral results, adults with ASD showed lower behavioralperformance in the 1-back task than TD adults, regardless of the stimulus category.Univariate analysis confirmed that the whole-body sensitive region in the bilateralLOTC was comparable between the ASD and TD groups. ANOSIM within the framework ofRSA revealed that activity in the LOTC was significantly clustered into threecategories: action effector body parts, face parts, and non-effector body parts. TheMantel test demonstrated that the dissimilarity maps of body parts in the LOTC weresignificantly similar between the TD and ASD groups. Furthermore, MVPA providedadditional evidence of the shared representational structure of body parts in theLOTC between the groups. However, body part representations in the bilateral LOTCshowed no relationship with individual traits in either group.

### 4.1 Behavioral performance

Behavioral analysis showed that the percentage of correct responses in the 1-backtask was lower in the ASD group than in the TD group. It is unlikely that thelow accuracy in the ASD group was attributable to a failure to complete thebutton-press task because the false alarm and response ratios for all stimuliwere comparable across both groups. There is abundant evidence that individualswith ASD show reduced performance compared to TD individuals in tasks thatrequire working memory (e.g., N-back tasks) (de Vries & Geurts, 2014;Geurts et al., 2004;Habib et al., 2019;Kenworthy et al., 2008;Williams et al., 2005,^2006^). As our study did not showa significant interaction between stimulus condition and group, the loweraccuracy observed in the ASD group may not be specific to the human body (facesor bodies). Therefore, the reduced response accuracy in our ASD group mayindicate that the working memory of visual images, regardless of object type, inindividuals with autism is lower than that in TD individuals, which isconsistent with previous studies (deVries & Geurts, 2014;Geurts et al., 2004;Habib et al., 2019;Kenworthy et al., 2008;Williams et al., 2005,^2006^).

### 4.2 LOTC function

RSA revealed that the TD and ASD groups showed comparable spatial representationsof each body part in the LOTC. In both groups, the spatial activity patterns ofthe bilateral LOTC were divided into three clusters: (1) action effector bodyparts (hands, feet, arms, and legs), (2) face parts (upper and lower faceparts), and (3) non-effector body parts (chest and waist). Although severalprevious fMRI studies have addressed EBA activation in adults with ASD (Okamoto et al., 2017), to thebest of our knowledge, no study has examined the body part representation inthis region using RSA. Thus, the present study provides novel evidence that thebody part representation in the LOTC of adults with ASD is similar to that in TDadults.

The main findings of this study were the significant division of representationsof body parts using RSA into three groups and the significant similarity betweenrepresentations of the TD and ASD groups. Univariate analysis revealed nosignificant differences in the bilateral whole-body-sensitive regions of theLOTC. However, using the Mantel test within the RSA framework, we found thatbody part representations in the LOTC were similar in TD adults and in thosewith ASD. Additionally, ANOSIM showed that, for both groups, bilateral LOTCspatial activation patterns were grouped into three clusters: (1) actioneffector body parts (hands, feet, arms, and legs), (2) face parts (upper andlower face), and (3) non-effector body parts (chest and waist). These resultsindicate that individuals with ASD show highly similar activation to TD adultsin terms of responsiveness of their neuronal populations to the body as a wholeand in their responsiveness to individual body parts. A previous study using apassive viewing task also revealed that adults with ASD showed a similaractivation in the higher visual cortex (EBA for body parts and fusiform facearea [FFA] for faces) to that of TD adults (Okamoto et al., 2017). However, when participantswere imitated by someone else, the activation of the left EBA was diminished inadults with ASD (Okamoto et al.,2014), suggesting that the EBA plays a role in higher-order cognitiveprocesses. This is further supported by studies showing a role for the EBA inthe recognition of social contingency during reciprocal imitation and the senseof agency (David et al., 2007,^2009^;Okamoto et al., 2014,^2017^). Similarly, several fMRIstudies in which participants were asked to perform a face-to-face task showed areduced FFA activation in adults with ASD (Critchley et al., 2000;Humphreys et al., 2008;Kleinhans et al., 2010;Pierce et al., 2001;Piggot et al., 2004;Pinkham et al., 2008;Schultz et al., 2000). Emotional facial expressionsare processed in a network between the “core system,” whichincludes the FFA, and the “extended system,” which includes theinferior parietal and frontal lobes associated with facial expressionrecognition, and the amygdala, insular cortex, and striatum associated withemotion recognition (Haxby &Gobbini, 2011). In contrast to previous studies, the body/faceviewing task used in the present study did not require higher-order cognitiveprocesses. The present results suggest that there is no difference in thelower-order processing of the whole body and the spatial representation of bodyparts between TD and ASD adults within the LOTC.

Unlike in adults, body part representation may differ between TD children andthose with ASD. A previous study showed that adults with ASD showed similaractivation in the EBA and FFA in TD adults, whereas children with ASD hadatypical activation in these areas. For example, we revealed that the EBA wassmaller in children with ASD than in TD children (Okamoto et al., 2017). Furthermore, when the amountand extent of neuronal activity in response to the human body increases fromchildhood to adulthood, the functional representation of these stimuli does notchange in terms of spatial encoding (Ross et al., 2014). Thus, there may be differences in therepresentation of body parts in the LOTC between individuals with ASD and TDindividuals until adolescence; however, these differences may disappear inadulthood. If this is the case, neural mechanisms underlying social difficultiesare different between adults and children with ASD and initial difference of theprocessing of bodies in children with ASD might contribute to future socialdifficulties.

### 4.3 Individual traits and representational geometry of body parts

Our previous study showed an association between body-parts representation in theLOTC and the sensory characteristics (sensory avoidance tendency), which arefrequently observed in ASD, in TD children and adolescents, although generalautistic traits were not associated with the LOTC (Okamoto et al., 2020). The findings lead to thepossibility that the pattern of body-parts representation in the LOTC leads tosensory-avoiding behaviors of children with ASD. In contrast, in the presentstudy, we did not find any correlation between the spatial organization of bodyparts in the LOTC and individual traits in adults with or without ASD. Althoughsome degree of sensory atypicality persists across ages in individuals with ASD(Leekam et al., 2007),atypicalities in the sensory profile of children with ASD tend to decrease withincreasing age (Baranek et al.,2019;Ben-Sasson et al.,2009). Behavioral studies have shown that body and face recognitiondifferences in ASD are lower in older individuals (Dowell et al., 2009;Serra et al., 1998;Strauss et al., 2012). Therefore, the observedabsence of an association between body part representation and sensorycharacteristics in adults with autism might be due to this age-related decreasein sensory atypicality, which supports the idea that the difference in body partrepresentation might be less in adulthood.

### 4.4 Limitation and future study

This study has five limitations. First, the sample size of the present study waslimited. Recently, it has been claimed that studies with small sample sizesusing resting-state fMRI and structural MRI have difficulties in detectingindividual differences (Marek et al.,2022). However, Marek et al. argue for the importance of small-sampleneuroimaging studies for clinical conditions using an induced-effects approach(such as task-related fMRI) (Marek etal., 2022). Task-related brain activation can improve the predictionof individual traits (Greene et al.,2018). However, a replication study with a larger sample couldprovide more robust knowledge. Second, as the participants were adults, it isunknown whether children with ASD showed similar or distinct results. Futurestudies involving a broader age range will provide important information forunderstanding the neural substrates of body perception from a developmentalperspective. Third, although the ASD-related measurements differ from those ofthe TD group, it remains unclear where they fall within the autism spectrum. Ina previous study, a meta-analysis of AQ and event-related potentials in thegeneral population, the P3b amplitude of event-related potentials during visualstimulus tasks was higher in accordance with the characteristics of autism(Mazer et al., 2024).Furthermore, a meta-analysis of fMRI revealed that individuals with ASD havemore activity in the extra-visual V2 cortex (BA18; equivalent to occipitalcortex) during visual processing than typical controls (Jassim et al., 2021). As theseare relatively simple tasks, we can provide a wider range of knowledge byconducting a meta-analysis including individuals with a wide range of ASD, suchas those with intellectual disabilities. Fourth, in the experimental frameworkof this fMRI study, it is difficult to examine brain regions involved inhigher-order processing of body parts, such as the middle temporal gyrus (MFG)and inferior frontal gyrus (IFG). In this study, we used a visual imagelocalizer (body parts vs. chairs) to focus on the LOTC. In order to examine RSAin other brain regions, it is necessary to set up an appropriate localizeraccording to their brain functions. For example, IPL is suitable for comparisonbetween action observation and execution (or resting) (de la Rosa et al., 2016;Koul et al., 2018). By setting appropriate localizersand examining higher brain regions, we can expect to gain further insights intothe processing of body parts in the brain. Finally, while the Mantel test inthis study revealed a statistically significant similarity in LOTCrepresentations between TD and ASD groups, the absence of observed groupdifferences means that concerns associated with a null effect—such aslimited statistical power and uncertainties regarding the positioning ofindividuals with ASD along the spectrum—remain unresolved. In order toaddress this issue, as mentioned in the first limitation, it will be necessaryin the future to consider whether the results of this study can be replicated byincreasing the number of TD and ASD individuals further.

## 5 Conclusion

The present study demonstrated that the spatial extent of the whole-body-sensitiveregion in the bilateral LOTC was similar between the ASD and TD groups. In addition,body part representation in the LOTC was similar across TD and ASD adults and wasorganized into three distinct clusters (i.e., action effector body parts,non-effector body parts, and face parts) in both groups. Furthermore, we found noassociation between body part representation clustering in the LOTC andautism-related individual traits. Considering the univariate analysis of each bodypart, the visual processing of body parts in the LOTC was comparable between adultswith and without autism. Alternatively, social difficulties in adults with ASD werenot due to different body-part representation in the LOCT and might be associatedwith a higher level of cognition.

## Supplementary Material

Supplementary Material

## Data Availability

Participant data used in this study cannot be made available for public access andare only available on request from researcher and optout.
