# Supplementary material for: Visual body part representation of the lateral occipitotemporal cortex in individuals with autism spectrum disorder: A univariate and multivariate fMRI study
Source: Imaging Neurosci (Camb). 2025 Jun 5;3:IMAG.a.24. doi: 10.1162/IMAG.a.24 (PMC12319863; doi:10.1162/IMAG.a.24)
Supplement: Supplementary Material [file imag.a.24_supp.pdf]

Supplemental Information

Figures

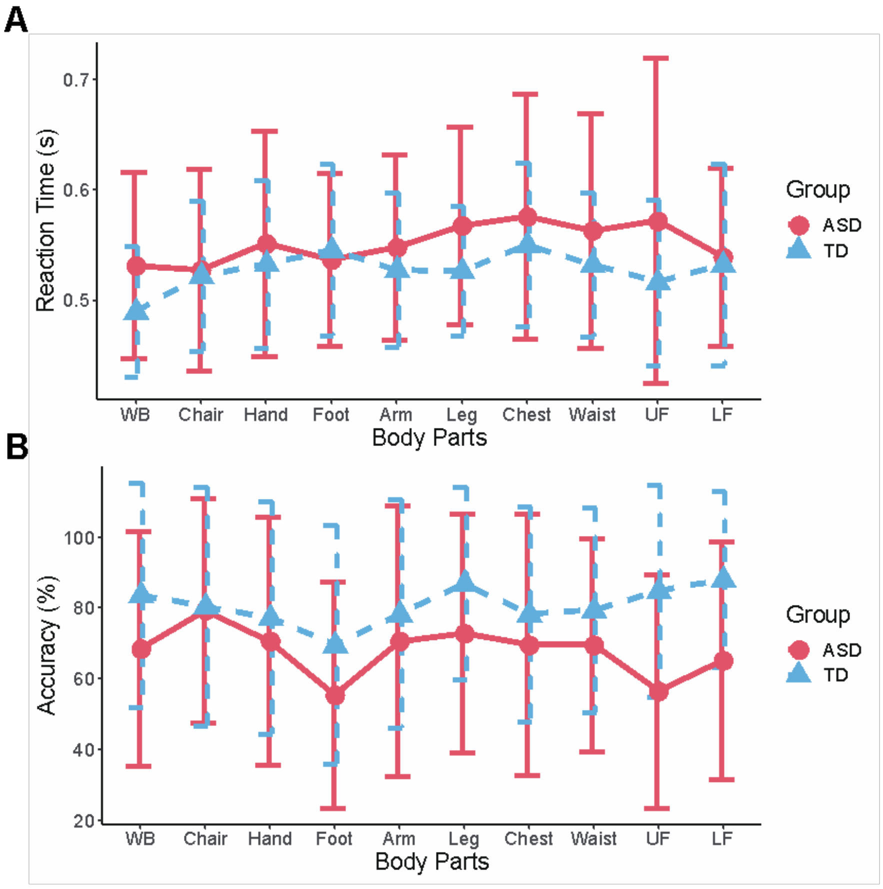

Figure S1. Line plots of behavioral performance of the 1-back task per body part for the TD and ASD groups. (A) Reaction time. (B) Accuracy. Error bars show the standard deviation. UF, upper face; LF, lower face; TD, typically developing; ASD, autism spectrum disorder.

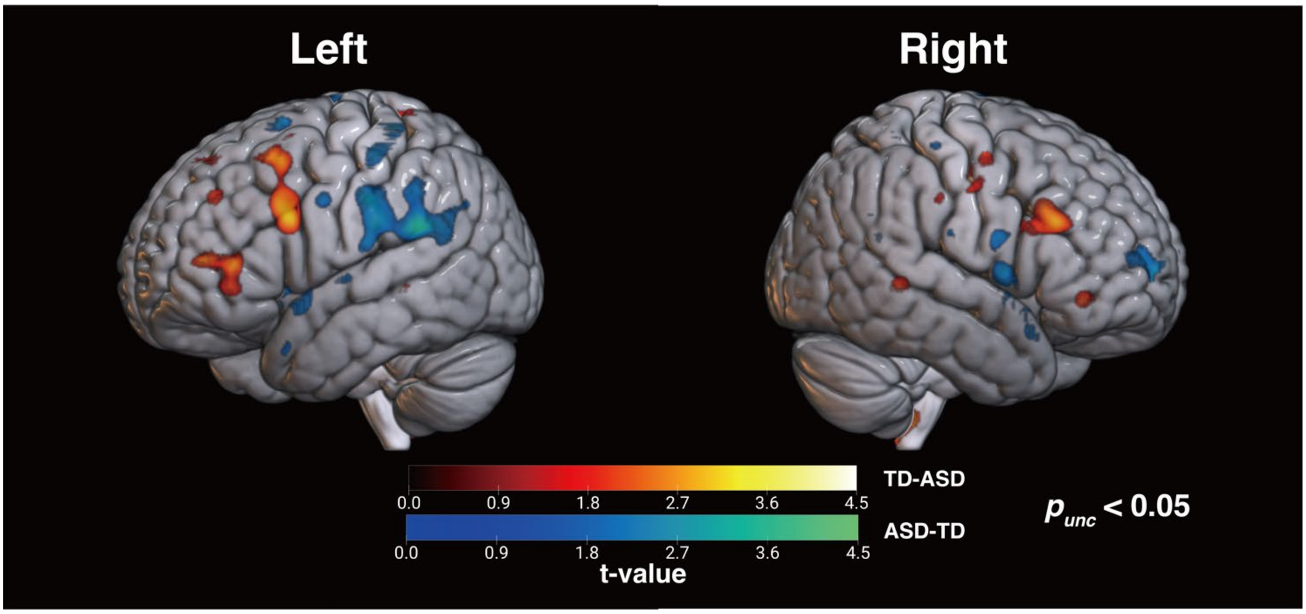

Figure S2: Surface-rendered t-map of the cluster regions showing fMRI signal changes for the whole-body condition compared to the chair condition during the 1-back task. Warm colors (red-yellow) regions indicate greater activation in TD relative to ASD, while cool colors (blue-green) regions indicate greater activation in ASD relative to TD. All clusters are displayed at an uncorrected threshold of $p < 0.05$.

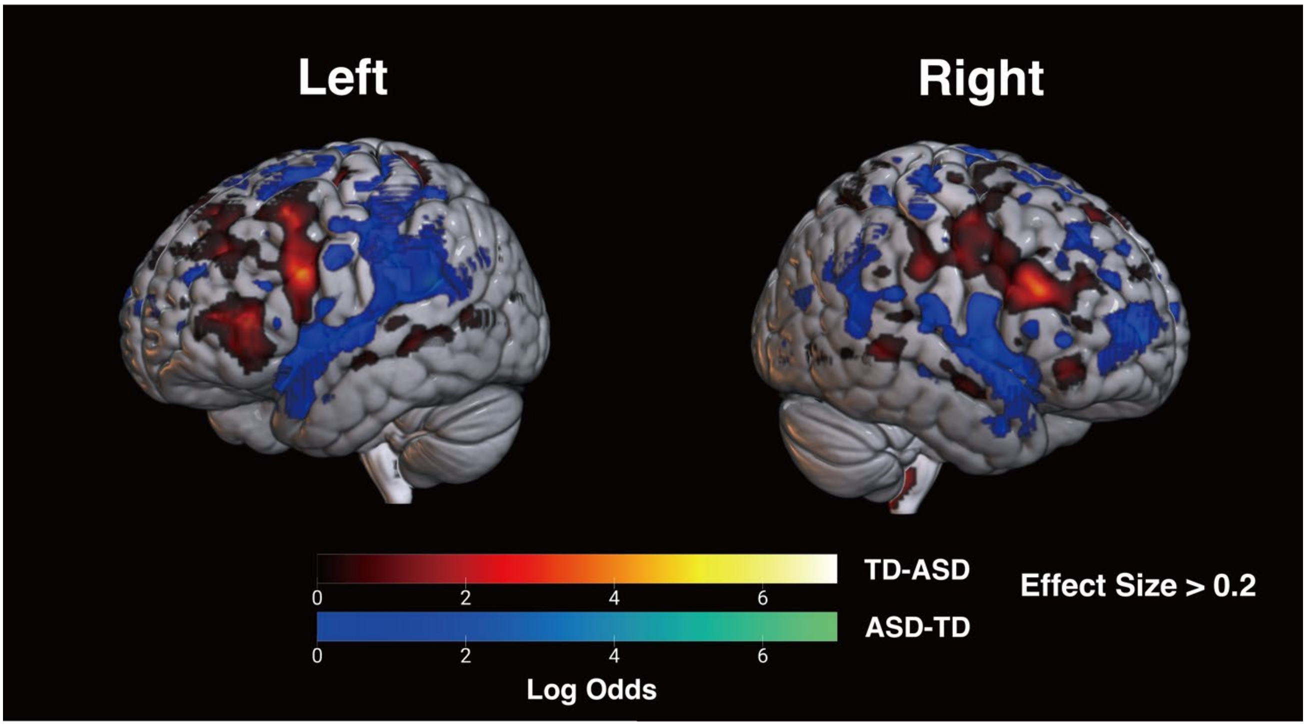

Figure S3: Surface-rendered Log-odd map of the cluster regions showing fMRI signal changes for the whole-body condition compared to the chair condition during the 1-back task, illustrating group differences between TD and those with ASD. Warm colors (red-yellow) regions indicate greater activation in TD relative to ASD, while cool colors (blue-green) regions indicate greater activation in ASD relative to TD. All clusters are displayed at an effect size threshold of 0.20.

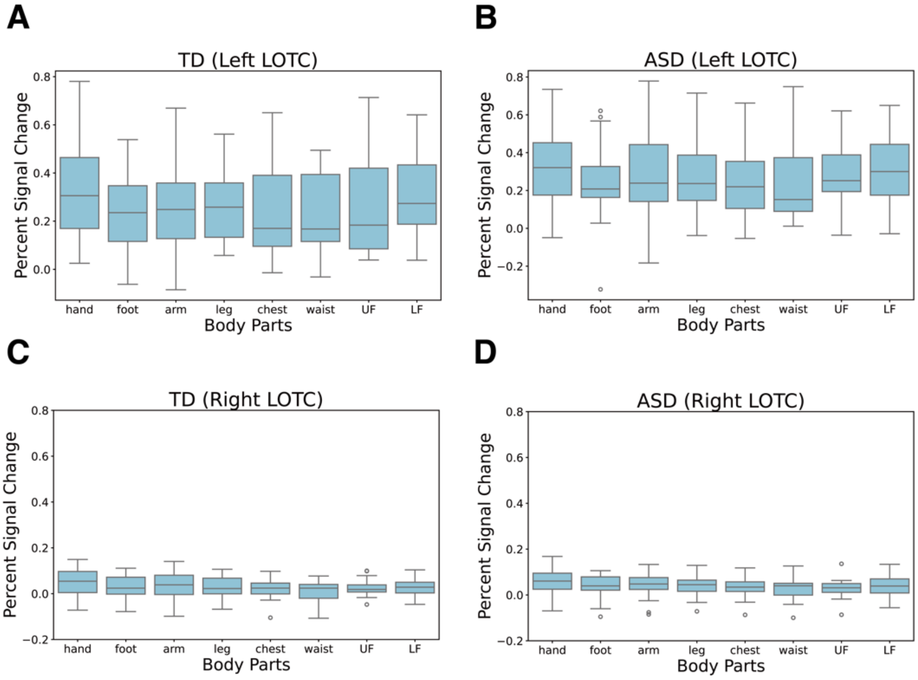

Figure S4: Box plots showing percent signal changes for each body part relative to baseline in the left and right LOTC. Panels (A) and (B) present data for the TD and ASD groups in the left LOTC, respectively, whereas (C) and (D) show data for the TD and ASD groups in the right LOTC, respectively.

Univariate fMRI analysis of the difference between TD and ASD in the classical (t-test) and Bayesian analysis

To explore potential group differences in brain responses, we generated t-maps for the contrast of whole-body versus chair stimuli, comparing TD and ASD participants (Figure S2). Notably, small clusters showing slightly greater activation in the TD group than in the ASD group were observed in frontal regions. However, since the maps are threshold at an uncorrected p-value ($p_{unc} < 0.05$), these findings should be interpreted with caution.

To complement the classical inference results, we conducted a Bayesian second-level analysis for the contrast of whole-body versus chair stimuli. In Bayesian inference, the log odds quantify the strength of evidence in favor of a given hypothesis. Specifically, SPM calculates the logarithm of the posterior odds for the hypothesis that the effect size exceeds a predefined threshold. Under a flat prior (i.e., prior odds = 1), this log odds is equivalent to the natural logarithm of the Bayes Factor, which compares the likelihood of the data under the alternative hypothesis (H1) versus the null hypothesis (H0). The resulting log odds maps are displayed in Figure S3, threshold at an effect size greater than 0.2. As this analysis was conducted with a relatively liberal effect size threshold for exploratory purposes, the results should be interpreted with caution and are not intended to support anatomical inferences.

Percent Signal Change for each body part

Using fMRI beta values, percent signal changes were computed and submitted to a two-factor ANOVA with Group (TD vs. ASD) and body part (hand, foot, arm, leg, chest, waist, upper face, lower face) as factors, conducted separately for the left and right LOTC (Figure S4). In both the left and right LOTC, there was a significant main effect of Body Part (left: $F_{7,308} = 6.983$, $p < 0.001$, $\eta_G^2 = 0.022$; right: $F_{7,308} = 15.745$, $p < 0.001$, $\eta_G^2 = 0.044$). Neither the main effect of Group (left: $F_{1,308} = 0.058$, $p = 0.812$, $\eta_G^2 = 0.001$; right: $F_{1,308} = 0.586$, $p = 0.448$, $\eta_G^2 = 0.012$) nor its interaction with Body Part (left: $F_{7,308} = 0.322$, $p = 0.944$, $\eta_G^2 = 0.001$; right: $F_{7,308} = 0.604$, $p = 0.753$, $\eta_G^2 = 0.002$) reached

significance.

Bonferroni-corrected post hoc comparisons ($p < 0.05$) in the left LOTC revealed that hand elicited greater responses than foot, waist, and chest, whereas waist exceeded lower face, and chest, foot, upper face, arm, and leg were all significantly lower than at least one other body part (e.g., chest and foot were lower than lower face, arm and leg were lower than waist, and upper face was also lower than lower face). In the right LOTC, hand elicited stronger responses than foot, waist, chest, and upper face, while waist, chest, and upper face were lower than lower face; in addition, leg and arm both exceeded waist. Taken together, these findings confirm body-part–specific activation in both LOTC regions, with no significant influence of or interaction with diagnostic group.

Bayesian analysis for TD/ASD body parts representations in left/right LOTC

We performed a Bayesian analysis for the Mantel test. Specifically, we fitted a Bayesian linear regression model in which the standardized distances from the TD dissimilarity matrix of body parts representation served as the predictor and the standardized distances from ASD similarity matrix of body parts representation as the response. The model was estimated via Markov chain Monte Carlo (MCMC) sampling using the {brms} package (version 2.22.0) in RStudio (version 024.04.2+764). We employed weakly informative priors for both the intercept and the slope (Normal(0,1)) and a Student-t prior (ν=3, mean=0, scale=1) for the residual variance. MCMC was run for 2,000 iterations across four chains, with the first 500 iterations of each chain discarded as burn-in.

In the left LOTC, the posterior mean of the slope was 0.91 (95% credible interval [0.75, 1.07]), suggesting a strong positive relationship between the TD and ASD dissimilarity matrices. To assess the evidence against the null hypothesis of β=0, we employed the Savage-Dickey density ratio method (Dickey, 1970), which compares the prior and posterior ordinates at the point β=0. The resulting Bayes factor (BF) was extremely large (effectively $BF>10^4$), providing decisive evidence against the null hypothesis. In addition, the posterior probability for β=0 was effectively zero, indicating that the data overwhelmingly favor a non-zero correlation.

In the right LOTC, the posterior mean of the slope was 0.96 (95% credible interval [0.85, 1.07]), suggesting a strong positive relationship between the TD and ASD dissimilarity matrices. The resulting Bayes factor (BF) was extremely large (effectively BF>104), providing decisive evidence against the null hypothesis. In addition, the posterior probability for β=0 was effectively zero, indicating that the data overwhelmingly favor a non-zero correlation.

Taken together, these results show that the null hypothesis of no association (β=0) can be rejected in a Bayesian framework, and that the TD and ASD body parts representations in the left/right LOTC exhibit similar.
